# A Prognostic Signature Consisting of Pyroptosis-Related Genes and SCAF11 for Predicting Immune Response in Breast Cancer

**DOI:** 10.3389/fmed.2022.882763

**Published:** 2022-05-12

**Authors:** Ling Chu, Qiaoli Yi, Yuanliang Yan, Jinwu Peng, Zhilan Li, Feng Jiang, Qingchun He, Lingzi Ouyang, Shangjun Wu, Chencheng Fu, Ying Liu, Zhijie Xu

**Affiliations:** ^1^Department of Pathology, The Third Xiangya Hospital, Central South University, Changsha, China; ^2^Department of Pharmacy, Xiangya Hospital, Central South University, Changsha, China; ^3^National Clinical Research Center for Geriatric Disorders, Xiangya Hospital, Central South University, Changsha, China; ^4^Department of Pathology, Xiangya Hospital, Central South University, Changsha, China; ^5^Department of Pathology, Xiangya Changde Hospital, Changde, China; ^6^Department of Emergency, Xiangya Hospital, Central South University, Changsha, China; ^7^Department of Emergency, Xiangya Changde Hospital, Changde, China

**Keywords:** pyroptosis, SCAF11, breast cancer, immune microenvironment, prognosis

## Abstract

Pyroptosis, characterized as an inflammasome-mediated cell death pathway, may be participated in tumorigenesis and progression. However, the underlying molecular function and mechanism of pyroptosis in BRCA remain unclear. In our study, we aimed to develop a prognostic signature in BRCA based on pyroptosis-associated genes. Data was downloaded from TCGA database, and then we screened 760 female BRCA samples and 104 normal breast tissues as the training set. Seven pyroptosis-related genes (CASP9, GPX4, IL18, NLRC4, SCAF11, TIRAP, and TNF) were identified as the pyroptosis-related prognostic model for BRCA using LASSO Cox regression. We subsequently tested the prognostic value of pyroptosis-associated gene signature in a validation set, GSE 20685. Time-dependent receiver operating characteristic analysis demonstrated the credible predictive capacity of this pyroptosis-associated gene signature. The area under the curves were 0.806 at 3 years, 0.787 at 5 years, 0.775 at 8 years, and 0.793 at 10 years in the training set, and 0.824 at 5 years, 0.808 at 8 years, and 0.790 at 10 years in the validation set. Furthermore, there are currently few data on SCAF11 regulating pyroptosis. To clarify this issue, we performed integrative bioinformatics and experimental analysis. Knocking down SCAF11 possessed an anti-cancer effect in terms of inhibiting cell viability and suppressing colony-formation in *in-vitro* functional assays. Meanwhile, the biological functions of SCAF11 in BRCA were further validated with several algorithms, such as Xiantao tool, LinkedOmics, GEPIA2, and TISIDB. These findings indicated that the expression of SCAF11 was significantly correlated with diverse tumor-infiltrating lymphocytes (TILs), including T central memory cell (Tcm), and type 2 T helper cell (Th2), etc. Functional enrichment analysis suggested that co-expression genes of SCAF11 primarily participated in inflammation and immune-related signaling pathways, such as oxidative phosphorylation, antimicrobial humoral response, and immunoglobulin complex. Moreover, SCAF11 expression was positively correlated with several immune checkpoints, including PD-L1, B7H3, and PDCD1LG2. Taken together, this study uncovered that pyroptosis-associated gene signature might be applied as an effective independent predictor in patients with BRCA. The pyroptosis-related gene SCAF11 might play potential roles in the regulation of immune microenvironment in BRCA.

## Introduction

Breast cancer (BRCA) is the most commonly diagnosed malignant tumor worldwide and the leading cause of cancer-related mortality in women, with an estimated 2.3 million new cases and 685 thousand deaths in 2020 (1). Risk factors for BRCA include estrogen exposure, aging, gene mutations, family history, and more (2). Currently, surgery, endocrine therapy, radiotherapy, chemotherapy, and human epidermal growth factor receptor 2 (HER2) targeted therapy are the main therapeutic modalities for BRCA (3, 4). Despite advances in primary prevention programs and therapeutic managements, the clinical outcomes for patients with advanced BRCA remain unfavorable and face significant clinical challenges (5). Therefore, novel and reliable screening programs are urgently needed to reduce BRCA mortality by early detection and effective treatment.

Pyroptosis is characterized as an inflammasome-mediated programed cell death modality that results in the cleavage of gasdermin D (GSDMD) or gasdermin E (GSDME) and secretion of proinflammatory cytokine IL-1β and IL-18 through pores in cell membrane, subsequently cell swelling, plasma membrane lysis, and eventually, cell death (6–8). Recently, an existing body of evidence suggests that pyroptosis may have impacts on the initiation, progression, invasion, and metastasis of cancer (9). In a recent study by Gao et al. GSDME-mediated pyroptosis could promote the development of colitis-associated colorectal cancer by releasing high-mobility group box protein 1 (HMGB1), a type of damage-associated molecular patterns (DAMPs), which participated in the tumorigenesis through the ERK1/2 pathway (10). Furthermore, combinations of BRAF inhibitors and MEK inhibitors in BRAF mutant melanoma promoted cleavage of GSDME and regulated the tumor immune microenvironment via pyroptosis (11). Xi's group revealed that GSDMD was significantly associated with CD8+ T cell in lung cancer cells (12). These studies implicated that pyroptosis may play a key role in antitumor immune responses. However, the underlying mechanism and function of pyroptosis in the immune microenvironment of BRCA are still unclear.

In this study, BRCA-associated molecular and clinical data were obtained from The Cancer Genome Atlas (TCGA) database. A prognostic risk model for female BRCA was developed based on 33 pyroptosis-related genes, and the prognostic value and clinical characteristics were then evaluated with this risk score model. In addition, we verified this prognostic model with dataset GSE 20685 acquired from Gene Expression Omnibus (GEO) database. The model consisted of seven pyroptosis-related genes, including CASP9, GPX4, IL18, NLRC4, SCAF11, TIRAP, and TNF. As there were few data on SCAF11 regulating pyroptosis, we further investigated the potential roles of SCAF11 in BRCA through experiments and bioinformatics analysis. The expression level of SCAF11 was up-regulated in BRCA, and knockdown of SCAF11 expression possessed an anti-cancer effect in inhibiting cell viability and suppressing of colony-formation. Functional enrichment analysis suggested that co-expression genes of SCAF11 primarily participated in inflammation and immune-associated signaling pathways. Moreover, SCAF11 expression was positively correlated with several immune checkpoints, such as programmed cell death ligand 1 (PD-L1), B7H3, and programmed cell death 1 ligand 2 (PDCD1LG2). In general, this study indicated that pyroptosis-associated gene signature might be an effective predictor in BRCA patients and may pave the way for the development of effective immunotherapy for BRCA.

## Materials and Methods

### Data Collection

**Figure 1:**
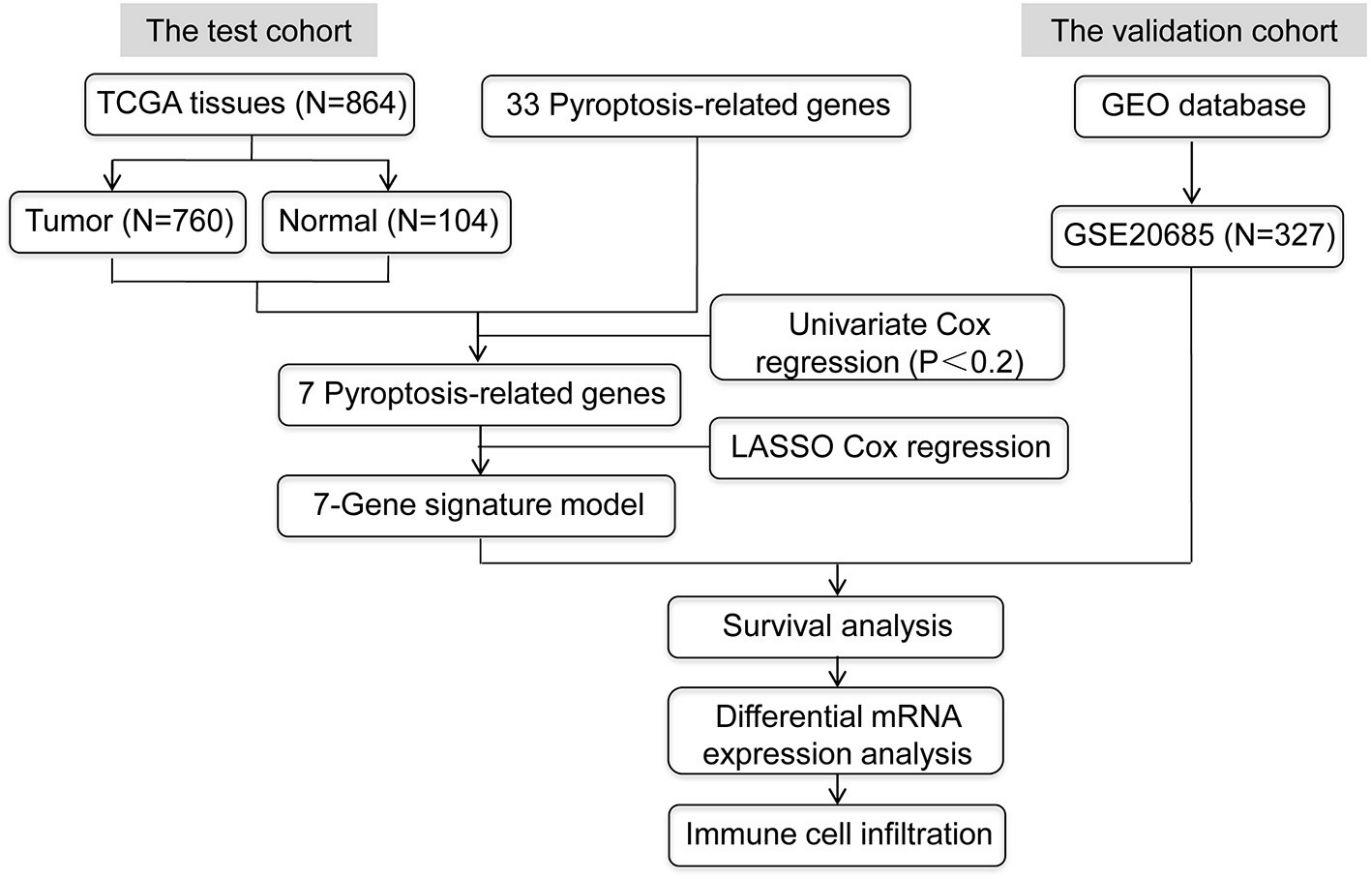
The detailed flow diagram of overall procedures in this study.

The mRNA expression levels from RNA-Seq and matching clinical data of TCGA-BRCA cohort were obtained from TCGA database (https://portal.gdc.cancer.gov/repository), and we screened 760 female BRCA samples and 104 normal breast tissues for further research as depicted in Figure 1. The gene annotation file (version GRCH38.84) was downloaded from the online Ensembl database (http://asia.ensembl.org/index.html). GSE 20685, acquired from the GEO database (https://www.ncbi.nlm.nih.gov/geo/query/acc.cgi?acc=GSE20685), was used as a validation dataset of the prognostic model. We performed the entire data analysis with the R statistical programming language (version 4.0.3).

### Identification of Pyroptosis-Related Genes

In this study, we extracted 33 pyroptosis-related genes from prior literature (Supplementary Table 1) (13–16). The prognostic values of these genes were then assessed by univariate Cox regression (*P* < 0.20). Eventually, 7 pyroptosis-related genes were selected to establish a prognostic risk model.

### Cell Culture and Reagents

Human BRCA cell lines, BT549 and T47D, were cultured in Roswell Park Memorial Institute (RPMI) 1640 (M210922, BasalMedia, China) supplemented with 10% fetal bovine serum (SA210623, Procell, China) and 1% penicillin-streptomycin (145394, Gibco, USA) in a humidified incubator at 37°C with 5% CO_2_. In addition, BT549 and T47D cells were transfected with SCAF11-targeted small interfering RNA (siRNA). The sequences of siRNAs are listed as follows: SiSCAF11-1 5′-CGAACATCCTAGATCTACA-3′; SiSCAF11-2 5′-GCAATCCATTTAACATTCA-3′.

### Western Blot

The cultured cells were collected and then lysed with IP lysis buffer supplemented with proteinase inhibitors. Equal amounts of total protein (30 μg) were loaded into each lane of 10% SDS–PAGE. Subsequently, proteins were transferred into PVDF membranes and blocked in 5% skimmed milk at RT for 2 h, and then incubated overnight at 4°C with primary antibodies in 5% BSA, followed by HRP-conjugated secondary antibody at RT for 1 h. Housekeep gene GAPDH served as an internal control. Primary antibodies included SCAF11 antibody (1:1,000, 28098-1-AP, proteintech) and GAPDH (1:10,000, 60004-1-Ig, proteintech). Finally, proteins were visualized using Immobilon Western chemiluminescent reagents (20020B6, Millipore, USA).

### Cell Counting Kit 8

BT549 and T47D cell lines were transfected with SCAF11-targeted siRNA or control siRNA for 48 h and then seeded in 96-well plates (2 × 10^3^ cells/well). At day 1, day 2, day 3, day 4, day 5, day 6, cell viability was assessed by CCK-8 assay (B34304, Bimake, USA) reading absorbance at 450 nm using a VICTOR X2 microplate reader (PerkinElmer, USA) according to the manufacturer's protocols.

### Colony Forming Assay

BT549 and T47D cells were transfected as previously described and then seeded in 6-well plates (10^3^ cells/well). After incubating for about 14 days, cell colonies were counted by staining with 0.3% w/v crystal violet/methanol.

### Construction and Validation of Pyroptosis-Related Gene Signature

Least absolute shrinkage and selection operator (LASSO) Cox regression with a 10-fold cross-validation was performed to determine the contributions of pyroptosis-related genes in survival prediction of TCGA-BRCA training cohort (17). We got 7 pyroptosis-related genes to establish the prognostic model with the “glmnet” R package. Risk score for each sample was calculated according to the linear summation of gene expression level multiplied by regression coefficient (β), and the formula was as follows: risk score = $\overset{n}{\underset{i=0}{\sum }}gene\;expression{\;}^{*}coefficient.$ Time-dependent receiver operating characteristic (ROC) curves, generated by the “TimeROC” R package, were used to assess the predictive power of pyroptosis-related gene signature over time. Furthermore, BRCA patients were divided into two different risk groups based on risk scores using the median as cutoff value. Kaplan–Meier survival analysis was performed using the “survival” R package to compare the differences in survival between low- and high-risk groups. Next, univariate and multivariate Cox regression analyses were performed to evaluate whether this pyroptosis-related gene signature showed good prognostic predictive power, independent of clinicopathological characteristics, including age and pathological TMN stage. Then, we established a prognostic nomogram including all independent prognostic parameters using a stepwise Cox regression model to predict the 3-, 5-, 8-, and 10-years overall survival (OS) of BRCA patients in the TCGA-BRCA cohort. Finally, we employed GSE 20685 as a validation dataset to test the applicability of this pyroptosis-related prognostic model by repeating the methods mentioned above.

### Function Enrichment and Immune Infiltrate Analysis

The online Xiantao tool (https://www.xiantao.love/products) is a comprehensive bioinformatics analysis portal for exploring differential expression, function enrichment analysis, interaction networks, and clinical significance across various cancers. We used Xiantao tool for differential expression of pyroptosis-related genes, gene set enrichment analysis (GSEA), and interactions between BRCA and immune system. Meanwhile, TISIDB (18) was also employed to cross-validate the interplay between SCAF11 and immune system, such as immune cells, and immunomodulators. Furthermore, the GEPIA2 database (19) was used to evaluate the correlation between SCAF11 and several immune monitoring sites in BRCA and the corresponding normal tissues.

LinkedOmics is a publicly available database providing multi-omics data across different cancers with three analytical modules, including LinkFinder, LinkInterpreter and LinkCompare (20). The heatmaps of the top 50 genes positively and negatively associated with SCAF11 were analyzed with the LinkFinder module. Spearman correlation test was used to perform correlation analysis.

### Statistical Analysis

Statistical analysis was performed using R statistical software, version 4.0.3, and its appropriate R packages. All fundamental experiments in our study were repeated at least three times. Values of *P* < 0.05 were regarded as statistically significant.

## Results

### Identification of Pyroptosis-Related Genes With Prognostic Values

Accumulating evidence shows that pyroptosis is involved in the initiation and development of cancer (8, 21). However, the underlying mechanism and function of pyroptosis in BRCA warrant further investigation. In this study, we screened a total of 864 samples (760 female BRCA patients and 104 normal breast tissues) from TCGA database. Furthermore, 33 pyroptosis-related genes were extracted from prior literature (Supplementary Table 1) (13–16), and the prognostic values of these genes were evaluated by univariate Cox regression (*P* < 0.20). The results showed that 7 pyroptosis-related genes, CASP9, GPX4, IL18, NLRC4, SCAF11, TIRAP and TNF, significantly correlated with prognosis (Supplementary Table 2). The detailed flow chart of this research was provided in Figure 1.

**Figure 2:**
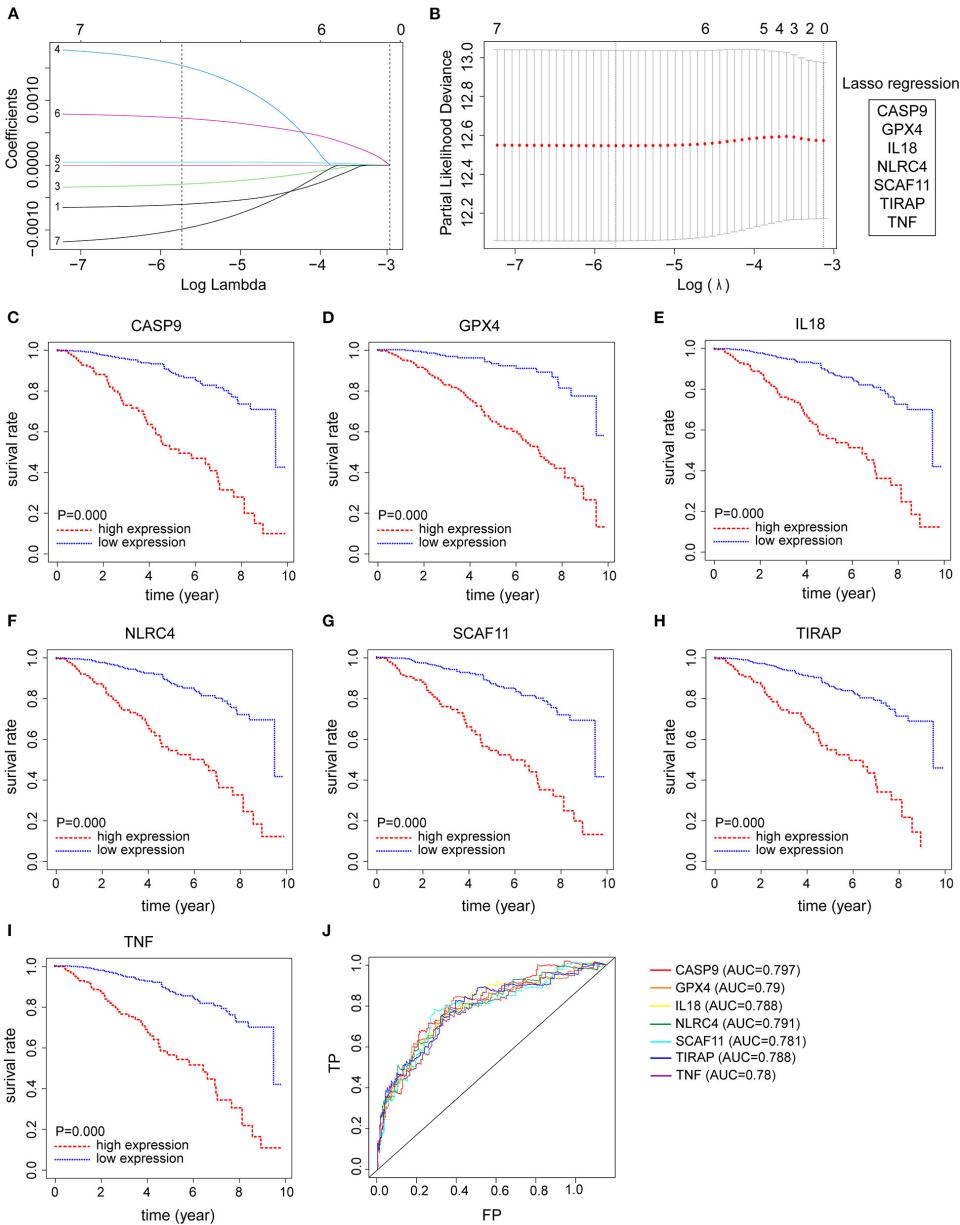
Identification of seven pyroptosis-related genes with prognostic power in breast cancer (BRCA) patients. **(A,B)** Seven pyroptosis-related genes with prognostic value were identified by LASSO Cox regression, including CASP9, GPX4, IL18, NLRC4, SCAF11, TIRAP, and TNF. **(C–I)** Kaplan-Meier survival analysis was employed to evaluate the prognostic capability of these candidate genes, respectively. **(J)** Time-dependent ROC curves were applied to assess the prognostic value of seven pyroptosis-related gene signature in the TCGA-BRCA cohort.

From the abovementioned 7 pyroptosis-related genes, all 7 genes were ultimately identified to be correlated to prognosis with LASSO Cox regression (Figures 2A,B). Kaplan–Meier survival analysis was plotted using the “survival” R package to evaluate the significance of 7 pyroptosis-related genes on the prognosis of patients with BRCA. As presented in Figures 2C–I, high expression levels of these candidate genes were all correlated with poor clinical outcome in BRCA patients. Additionally, time-dependent ROC analyses for predictive power of these selected genes acquired area under curve (AUC) values of 0.797 for CASP9, 0.790 for GPX4, 0.788 for IL18, 0.791 for NLRC4, 0.781 for SCAF11, 0.788 for TIRAP, 0.780 for TNF (Figure 2J).

### Construction of Pyroptosis-Related Genes Signature

**Figure 3:**
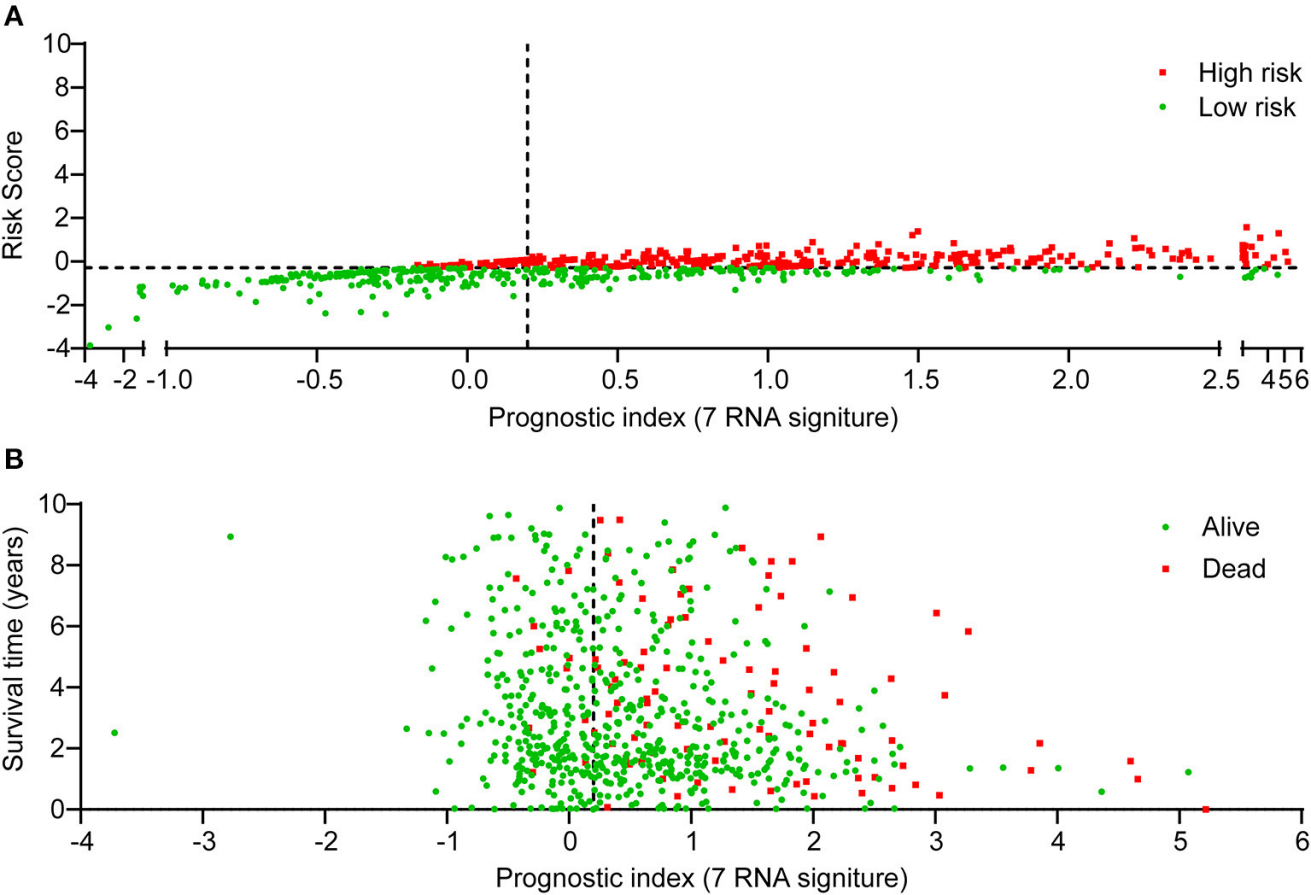
The distribution of BRCA patients according to the risk score for pyroptosis-related gene signature. The 7-gene signature risk score ranged from −3.865 to 1.572. The median value (−0.291) was used as cutoff point. Scatter plots were employed to indicate the risk score **(A)** and survival outcome **(B)** of each BRCA patient. “Dead” and “Alive” were represented by red and green dots in **(B)**, respectively.

Risk score for the expression of 7 pyroptosis-related genes (risk score = −9.715e-04 ^*^ CASP9 - 9.623e-06 ^*^ GPX4 - 2.186e-04 ^*^ IL18 + 2.604e-03 ^*^ NLRC4 + 2.675e-05 ^*^ SCAF11 + 6.579e-04 ^*^ TIRAP - 1.350e-03 ^*^ TNF) for each sample was calculated with the “glmnet” R package (Supplementary Table 3). Next, the median risk score was used as the cutoff value. Based on the cutoff point of risk score, patients of TCGA-BRCA cohort were divided into low-risk and high-risk groups. Figure 3A showed the scatter plot distribution between risk score and prognostic index, and Figure 3B presented the scatter plot distribution between survival status of each BRCA patient and prognostic index. As we can see from Figure 3B, the majority of death cases were distributed in high-risk group. Taken together, these findings indicated that pyroptosis-related seven-gene signature exhibited its potential as the prognostic signature for BRCA patients.

### The Prognostic Value of Pyroptosis-Related Genes Signature

**Figure 4:**
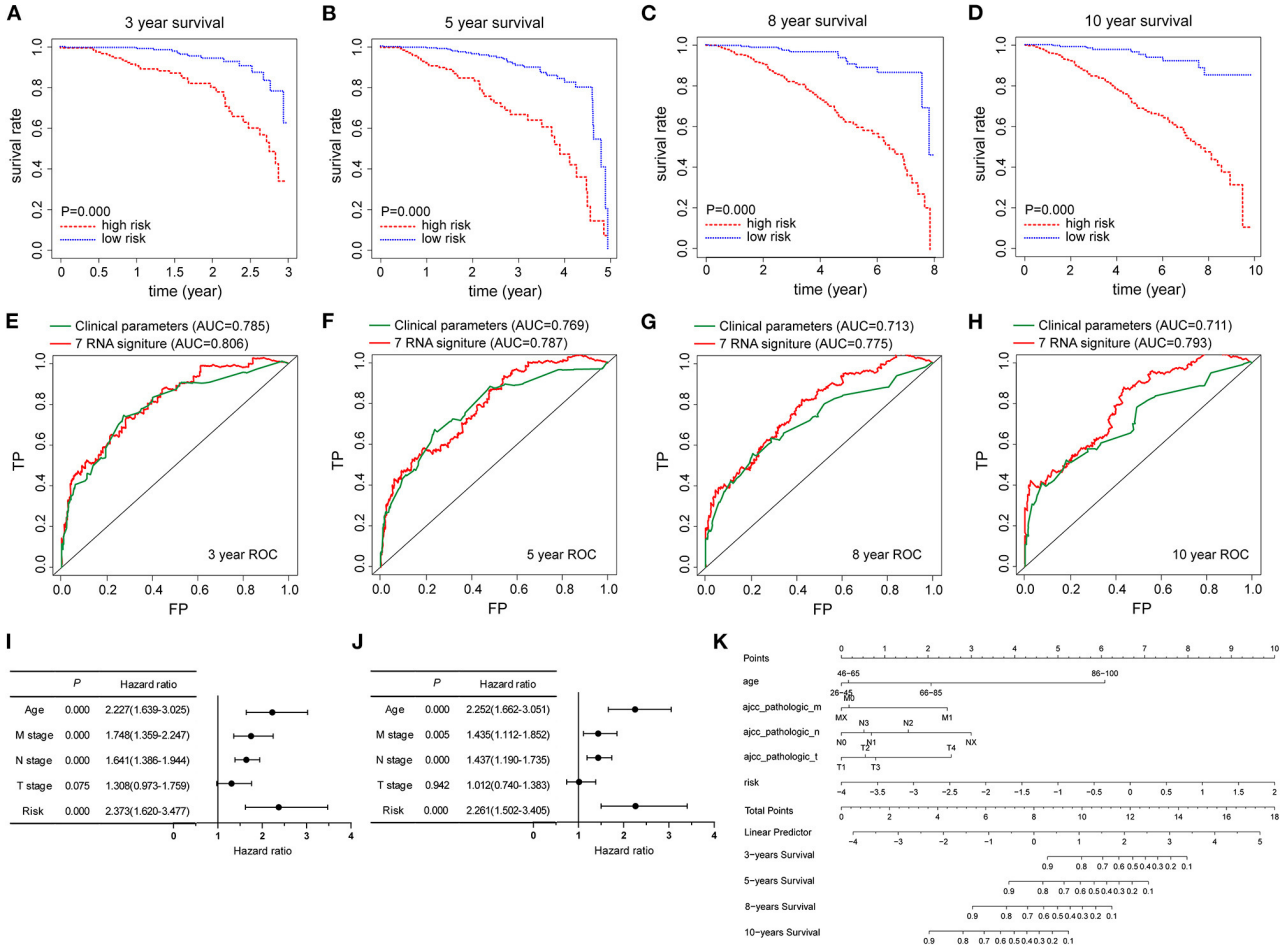
The prognostic capability of pyroptosis-related gene signature in TCGA-BRCA cohort. **(A–D)** Kaplan-Meier survival curves were employed to compare the differences in survival rates at 3-, 5-, 8-, and 10-years between high-risk and low-risk groups based on the risk score model, respectively. **(E–H)** Time-dependent ROC curves were used to assess the prognostic value of the risk signature at 3-, 5-, 8- and 10-years, respectively. **(I,J)** Forest plots of univariate and multivariate Cox regression analysis in TCGA-BRCA cohort, including clinicopathological factors and the risk score model. **(K)** Nomogram combined with the risk signature and clinicopathological factors. Nomogram to predict the 3-, 5-, 8-, and 10-year survival probabilities of BRCA patients in the TCGA-BRCA cohort based on various clinicopathologic factors, including 7-gene signature risk score, age and tumor stage.

Kaplan–Meier survival curves were presented in Figures 4A–D, the 3-, 5-, 8-, and 10-year survival rates of TCGA-BRCA patients in the high-risk group were remarkably lower than those in the low-risk group. Furthermore, the time-dependent ROC analyses showed the predictive power of risk score in prognosis. The AUCs of 3-, 5-, 8-, and 10-year were 0.806, 0.787, 0.775, and 0.793, respectively (Figures 4E–H). Next, in univariate Cox regression analysis, age, N stage, M stage, and risk score showed an association with prognosis in TCGA-BRCA cohort (Figure 4I). Factors identified by univariate Cox regression analysis were subsequently analyzed by using multivariate Cox regression, and the results suggested that age, N stage, M stage, and the prognostic model were independent prognostic predictors (Figure 4J). To make the signature based on pyroptosis-related genes more convenient in clinical application, a nomogram incorporating these predictors was constructed to explore the validity of pyroptosis-related gene signature in predicting the 3-, 5-, 8-, and 10-year survival rate in the TCGA-BRCA cohort. As provided in Figure 4K, risk score model performed the best weight among all relevant covariates.

**Figure 5:**
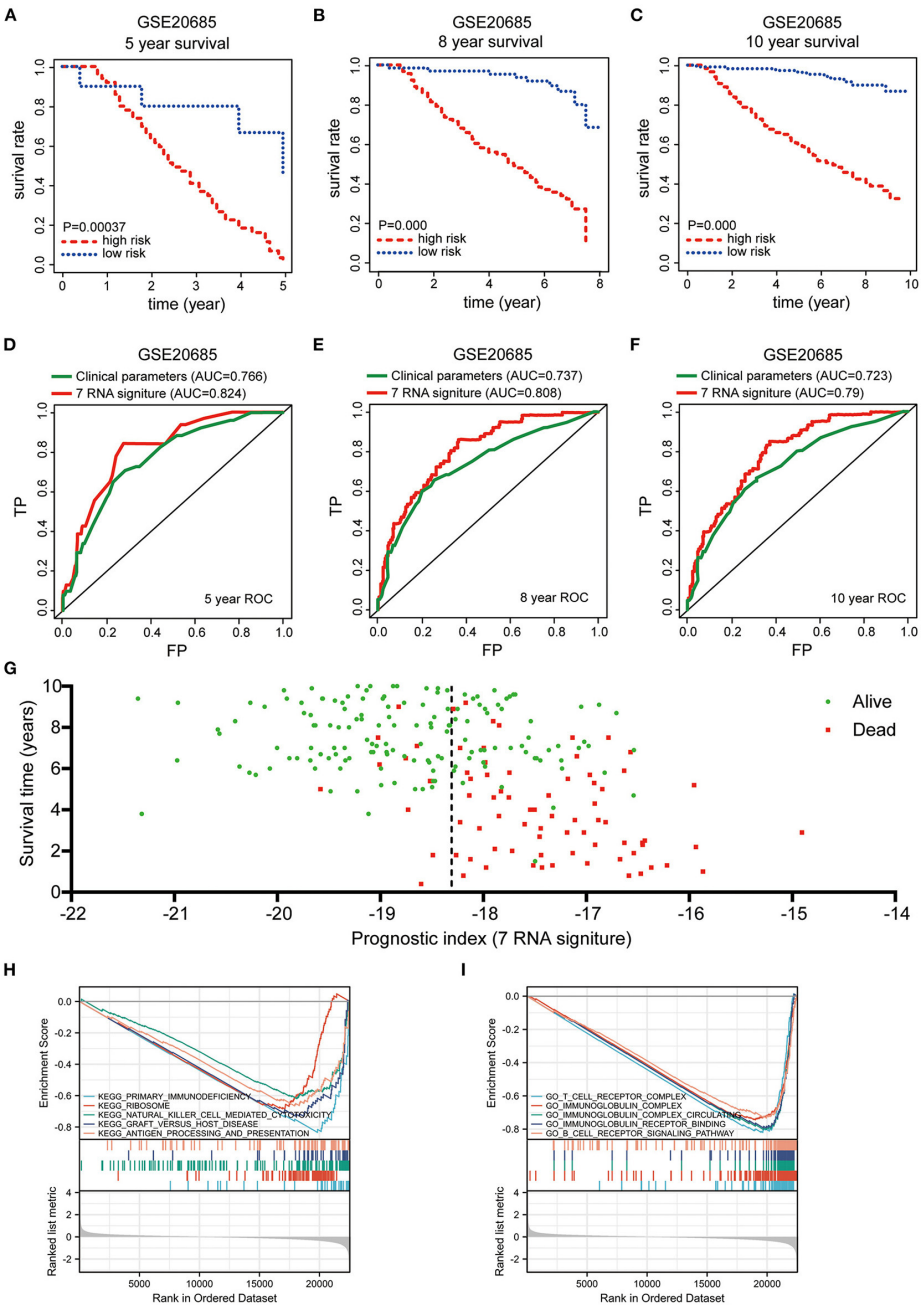
Validation the prognostic value of pyroptosis-related gene signature in GSE 20685 and GSEA enrichment analysis for pyroptosis-related gene signature in TCGA-BRCA. **(A–C)** Kaplan-Meier survival curves were employed to validate the prognostic power of pyroptosis-related gene signature in GSE 20685 at 5-, 8-, and 10-years survival rates, respectively. **(D–F)** Time-dependent ROC curves were used to assess the prognostic value of the risk signature in the GSE 20685 dataset at 5-, 8-, and 10-years, respectively. **(G)** Scatter plot were used to indicate the survival status of each Breast cancer case from GSE 20685. “Dead” and “Alive” were represented by red and green dots in **(G)**, respectively. **(H)** KEGG and **(I)** GO pathway enrichment analysis for pyroptosis-related gene signature between low- and high-risk group in TCGA-BRCA cohort.

In order to test this gene signature in external datasets, we employed GSE 20685 as the validation dataset, which contains gene expression profiles and clinical characteristics of 327 BRCA samples, and repeated the abovementioned data analyses. Consistent with the findings from TCGA-BRCA cohort, Kaplan–Meier survival curves showed that BRCA patients with high-risk scores harbored a poorer prognosis (Figures 5A–C). The risk score had favorable prognostic prediction value with the AUCs of 0.824, 0.808, and 0.79 at 5-, 8-, and 10-year, respectively (Figures 5D–F). Moreover, survival time scatter plot revealed that as the prognostic index rose, the cancer deaths increased, and the survival time shortened (Figure 5G). All these results revealed that this pyroptosis-related seven-gene signature exhibited its superior specificity and sensitivity to predict the clinical outcomes of BRCA patients. To further explore the potential differences in biological function between high- and low-risk group, the GSEA functional enrichment analysis indicated that some immune and inflammation-related KEGG pathways were inhibited in the high-risk group, including primary immunodeficiency, natural killer cell mediated cytotoxicity, and graft vs. host disease (Figure 5H). The suppressed GO pathways in high-risk group were mainly distributed in immune-related pathways, such as T cell receptor complex, immunoglobulin complex, immunoglobulin complex circulating, immunoglobulin receptor binding, and B cell receptor signaling pathway (Figure 5I). Taken together, the GSEA functional enrichment analysis results suggested the biological significance of this established pyroptosis-related gene signature in inflammation and immune regulation. These findings indicated that this novel pyroptosis-related gene signature could be served as a reliable independent prognostic factor in BRCA patients.

### SCAF11 Knockdown Inhibited Cell Proliferation in BRCA

**Figure 6:**
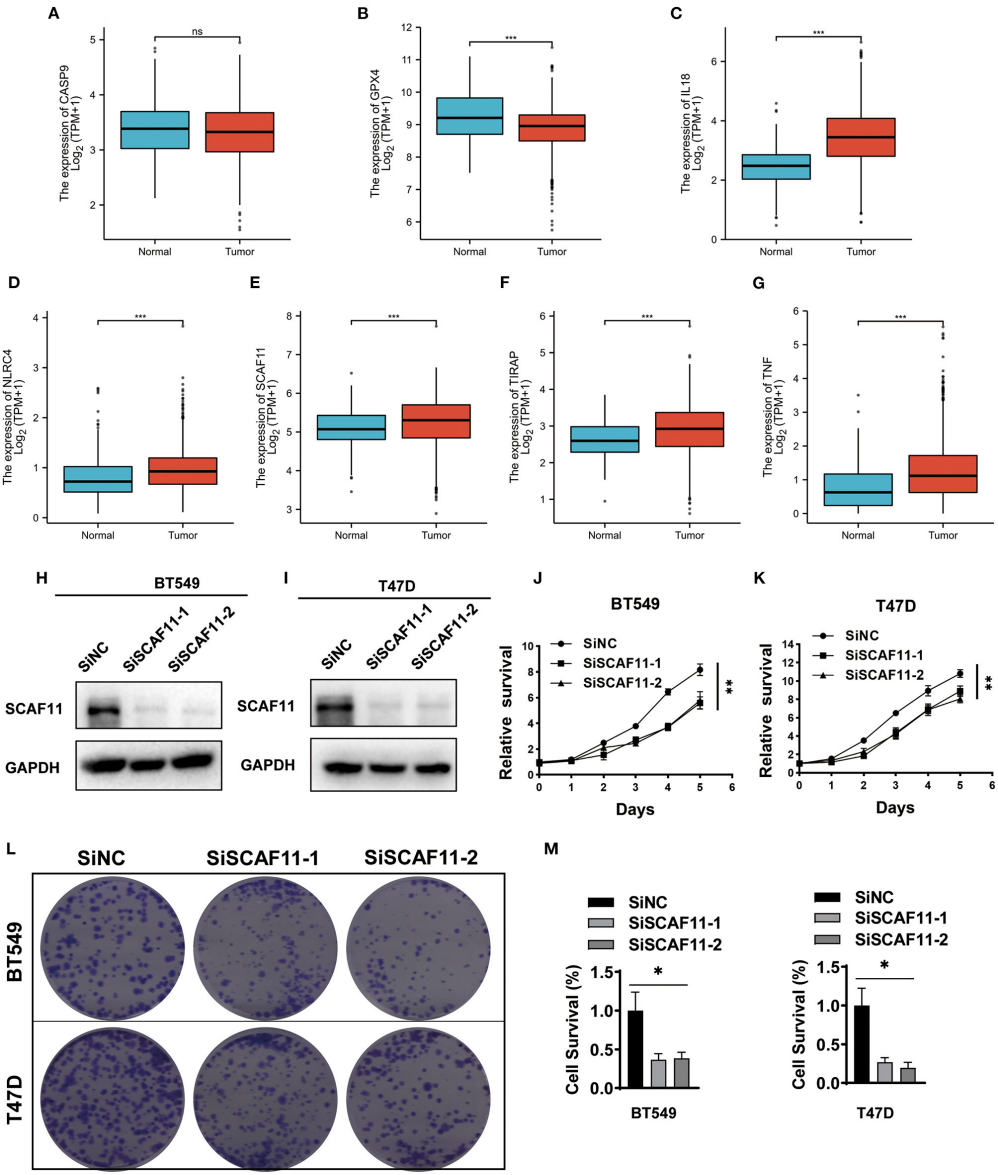
The expression of seven pyroptosis-related genes in BRCA tissues and SCAF11 knockdown inhibited the cell proliferation. **(A–G)** Differential mRNA expression analysis of the seven pyroptosis-related genes in BRCA samples and normal breast tissues. The Xiantao tool was used to perform the expression profiles. **(H,I)** Knockdown efficacy of siRNA against SCAF11 in two human breast cancer cell lines, BT549 and T47D, was analyzed by western blotting. **(J,K)** CCK-8 assay indicated the effects of SCAF11 knockdown on cell proliferation rate of BT549 and T47D. **(L,M)** SCAF11 knockdown inhibited the cell colony formation rate in BT549 and T47D cell lines. *p < 0.05, **p < 0.01, ***p < 0.001, ns: no statistical significance.

Differential expression analysis was carried out by comparing BRCA tumor samples to normal breast tissues with the online Xiantao tool. As shown in Figures 6A–G, pyroptosis-related genes including IL18, NLRC4, SCAF11, TIRAP, and TNF were highly expressed in BRCA patients, consistent with our finding that high expression of these genes was associated with poor outcome. However, no experimental information is currently available on the function of SCAF11 in the oncogenesis and progression of BRCA. Further studies are necessary to be conducted in order to clarify this issue. Given the high expression of SCAF11 in BRCA tumor tissues, we knocked down SCAF11 expression with siRNAs in two BRCA cell lines, BT549 and T47D, and SCAF11 knockdown efficacy was verified by immunoblotting (Figures 6H,I). Moreover, CCK-8 and colony formation assay indicated that SCAF11 knockdown by siRNAs significantly attenuated cell proliferation and inhibited clone forming ability in BT549 and T47D (Figures 6J–M). These findings suggested that SCAF11 may conduct its tumor-promoting effect in the development of BRCA.

### SCAF11 Co-expression Network and GSEA Enrichment in BRCA

**Figure 7:**
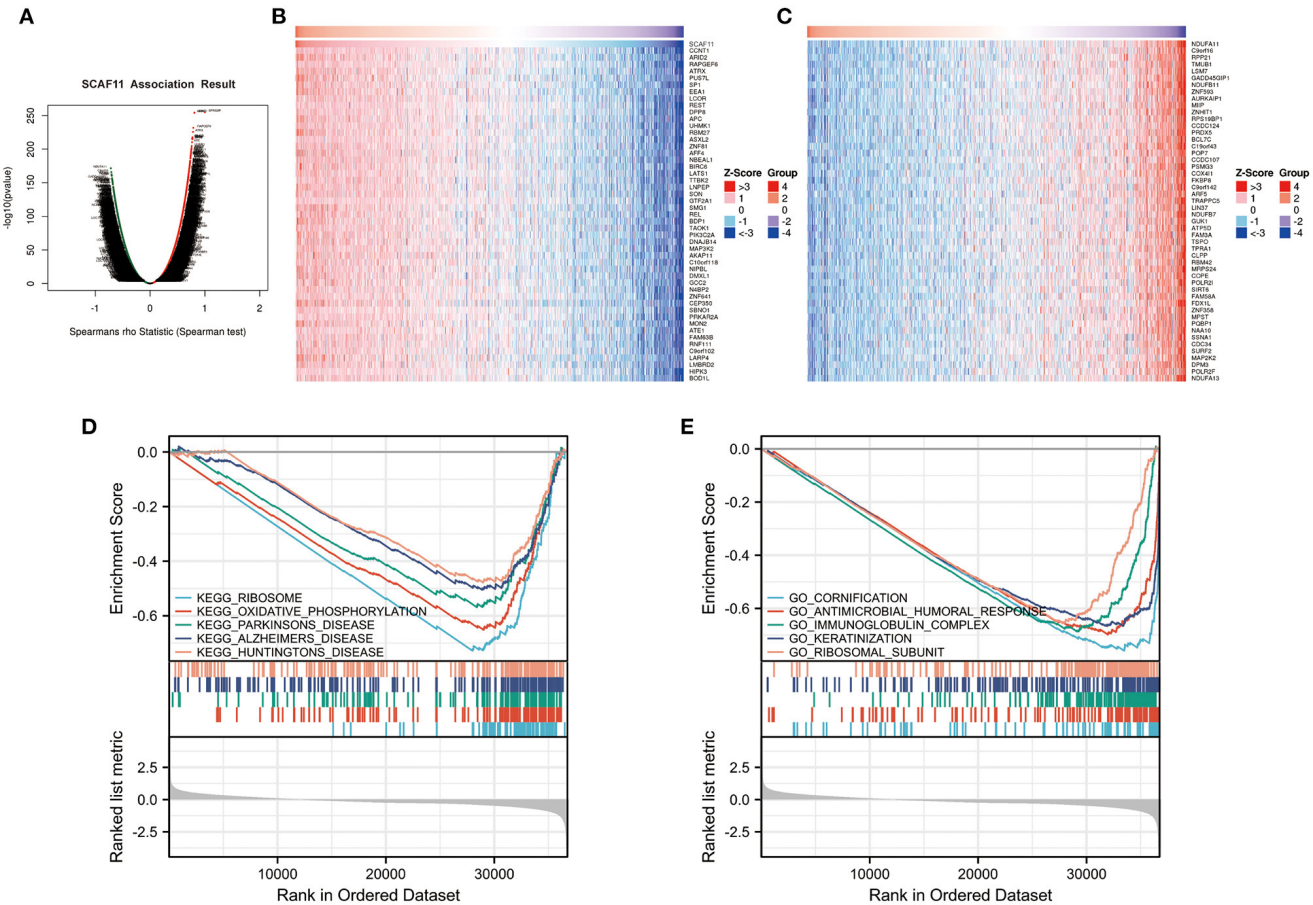
The co-expression genes with SCAF11 from the LinkedOmics database and GSEA enrichment analysis from Xiantao tool. **(A)** Volcano plot showing genes significantly associated with SCAF11 by Spearman test. **(B,C)** Heatmaps of the top 50 genes positively and negatively correlated with SCAF11, respectively. **(D,E)** KEGG and GO pathway analysis.

To investigate the biological roles of SCAF11 in BRCA, the LinkFinder module of the LinkedOmics database was used to conduct the co-expression pattern of SCAF11. As presented in Figure 7A, SCAF11 was positively associated with 8,000 genes (red dots), and negatively correlated with 7,577 genes (green dots). Figures 7B,C exhibited the heatmaps of the top 50 genes positively or negatively correlated with SCAF11, respectively. In addition, we applied KEGG and GO enrichment analysis to explore the functional enrichment of co-expression genes of SCAF11 in BRCA. KEGG pathway analysis indicated that these genes mainly participated in oxidative phosphorylation and some neurodegenerative diseases, such as parkinson's disease, alzheimer's disease, and huntington's disease (Figure 7D). GO enrichment analysis revealed that these genes may be involved in regulating inflammation and immune-related pathways, such as antimicrobial humoral response and immunoglobulin complex (Figure 7E). GSEA enrichment analysis suggested the biological significance of SCAF11 in inflammation and immune regulation.

Furthermore, we wanted to explore whether other pyroptosis-related genes identified as pathologically expressed in BRCA have biological functions similar to SCAF11. As shown in Supplementary Figure 1, KEGG pathway analysis indicated that co-expression genes of IL18 mainly participated in immune and inflammation-related pathways, such as natural killer cell mediated cytotoxicity, and graft vs. host disease. GO enrichment analysis revealed that these genes mainly involved in immune-related pathways, such as immunoglobulin complex, T cell receptor complex, immunoglobulin production, lymphocyte mediated immunity and B cell mediated immunity. Also, other pyroptosis-related genes, such as NLRC4, TIRAP and TNF, were found to be associated with immune and inflammation-related pathways.

### Relationship Between SCAF11 and Immune Molecules

**Figure 8:**
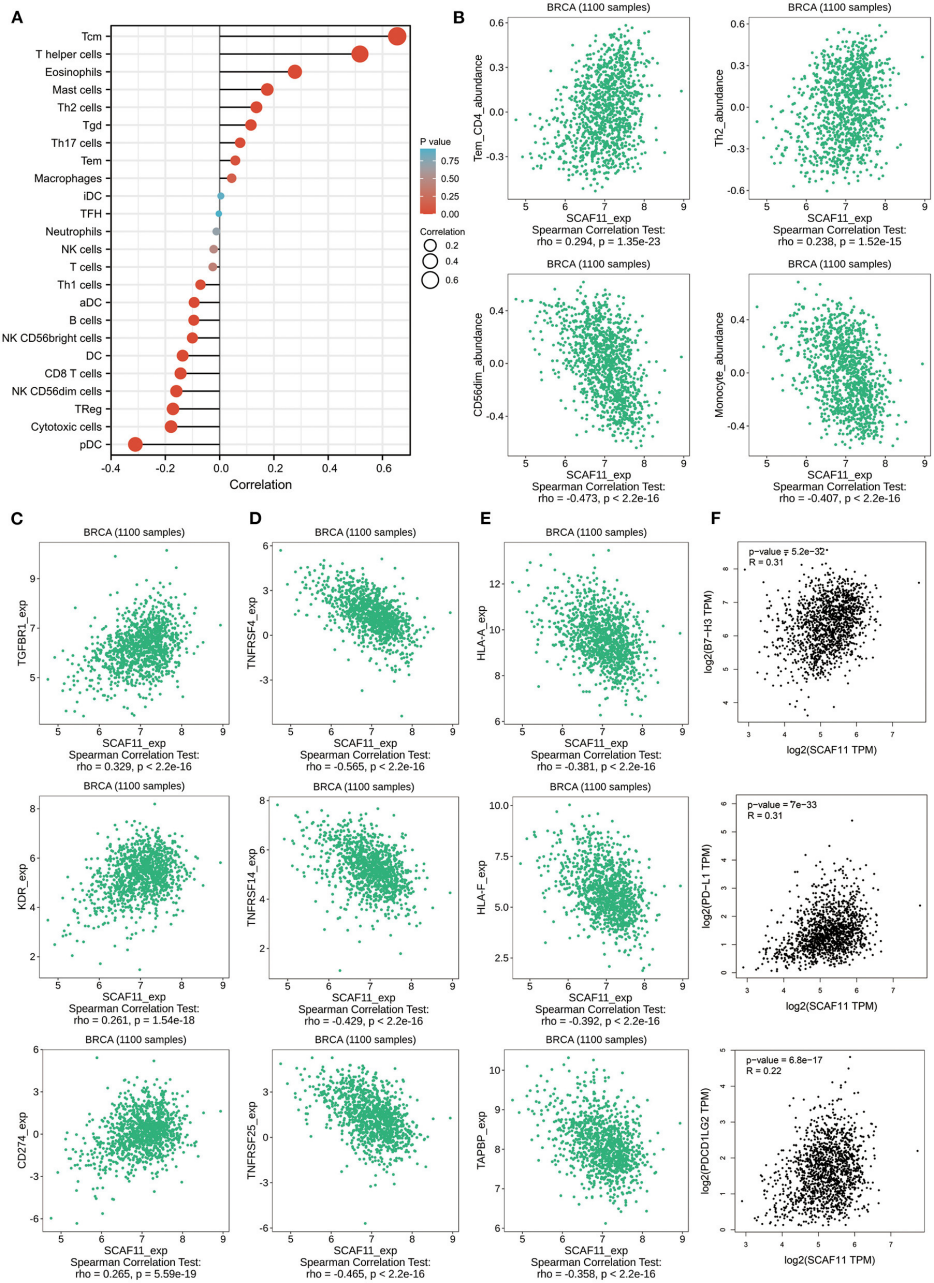
The relationships between immune cell infiltration with SCAF11. **(A)** Immune infiltration cells associated with SCAF11 by Xiantao tool. **(B)** Validations of partial lymphocytes by the TISIDB database. Associations of SCAF11 expression with immunoinhibitors **(C)**, immunostimulators **(D)**, and MHC molecules **(E)**. **(F)** The association between SCAF11 with PD-L1, B7-H3, and PDCD1LG2.

We further analyzed the association between SCAF11 expression level and tumor-infiltrating immune cells in BRCA with Xiantao Tool. There were apparently positive correlations between SCAF11 expression level and several tumor-infiltrating lymphocytes (TILs), including T central memory cell (Tcm), and type 2 T helper cell (Th2) (Figure 8A). we also found significantly negative correlations between SCAF11 expression and other TILs, like CD56dim natural killer cell (CD56dim), and monocyte. Similar results were seen in TISIDB database by analyzing immune-associated 28 TIL types (Figure 8B).

We further explored the correlations between SCAF11 expression and other immune signatures, such as immunoinhibitors, immunostimulators, and major histocompatibility complex (MHC) molecules, using the TISIDB database. As provided in Figure 8C, the expression level of SCAF11 was positively correlated with immunoinhibitors, including transforming growth factor beta receptor 1 (TGFBR1), kinase insert domain receptor (KDR), and cluster of differentiation 274 (CD274). Figure 8D exhibited the negative correlation between SCAF11 expression and immunostimulators, such as TNF receptor superfamily member 4 (TNFRSF4), TNF receptor superfamily member 14 (TNFRSF14), and TNF receptor superfamily member 25 (TNFRSF25). Figure 8E revealed the relationship between SCAF11 expression and MHC molecules, including major histocompatibility complex, class I, A (HLA-A), major histocompatibility complex, class I, F (HLA-F), and TAP binding protein (TAPBP). These findings indicated that TILs and immune molecules infiltration might play a key role in the prognosis of patients with BRCA. Given the importance of clinical application of immune checkpoint blockade therapy in BRCA patients, we then explored the association between SCAF11 expression and several immune checkpoints. Figure 8F showed that the expression level of SCAF11 was positively correlated with programmed cell death ligand 1 (PD-L1), B7H3 (also known as CD276), and programmed cell death 1 ligand 2 (PDCD1LG2). In general, these data suggested that pyroptosis-related gene signature might influence the response to immunotherapy in BRCA patients.

## Discussion

In this study, we established a unique prognostic evaluation model by integrating traditional clinicopathological parameters and risk score based on pyroptosis-related genes in a large-scale BRCA cohort, including TCGA-BRCA as a training dataset and GSE 20685 as a test dataset, and demonstrated its sensitivity and specificity. Furthermore, the relationship between SCAF11 and immune microenvironment indicated that it may be responsible for the poor prognosis for BRCA patients. The nomogram established a link between risk score and clinical outcome. Factors affecting the prognosis of BRCA patients were considered from multiple perspectives, laying a firm foundation for clinical management and treatment.

Pyroptosis has been recognized as a unique programmed cell death modality executed by the pore-forming gasdermin family, coexisting immune and inflammatory responses (15, 22). Pyroptosis was first discovered in macrophages infected with *Salmonella typhimurium* (23). In addition to infectious diseases, later it was found to be involved in cardiovascular and neurological diseases, especially atherosclerosis, myocardial infarction, and neurodegenerative diseases (24, 25). Moreover, inflammasome-activated pyroptosis and pyroptosis-produced cytokines play a predominant role in tumorigenesis, progression, and immune regulation (26). Previous studies convincingly demonstrated that GSDME mRNA methylation resulted in GSDME downregulation in various cancers, and downregulated GSDME levels were related with decreased survival rate in BRCA. GSDME repressed tumor growth by enhancing anti-tumor immune response, especially the enhanced phagocytosis of tumor-associated macrophages and the number and functions of CD8^+^ T lymphocytes and tumor-infiltrating natural killer cells (27). However, as a form of proinflammatory cell death mechanism, pyroptosis can promote tumor growth by forming a microenvironment suitable for tumor cell growth (15). Therefore, our study focused on exploring the interplay pattern of pyroptosis in BRCA and constructed a pyroptosis-associated prognostic predictive model.

We finally identified 7 pyroptosis-related genes in this research, and some of which were reported to be involved in BRCA or other cancer types. Among them, SCAF11 (SR-related CTD associated factor 11), also known as splicing factor, arginine/serine-rich 2-interacting protein (SFRS2IP), is a member of human SR (Ser/Arg-rich) superfamily. Few data are currently available on the regulation of pyroptosis by SCAF11 (28, 29). *In vitro* experiments indicated that SCAF11 was highly expressed in breast tumor cell lines. The high expression of SCAF11 was correlated with poor prognosis in BRCA patients, and SCAF11 knockdown by siRNAs significantly suppressed cell proliferation and colony formation in BT549 and T47D BRCA cell lines. Furthermore, GSEA analysis revealed that co-expression genes of SCAF11 mainly participated in inflammation and immune-associated pathways, such as antimicrobial humoral response, and immunoglobulin complex. SCAF11 expression was positively correlated with several immune checkpoints, including PD-L1, B7H3, and PDCD1LG2. Together, these findings indicated that SCAF11 may be a pro-oncogenic factor associated with the regulation of pyroptosis-related pathway in BRCA and inhibition of SCAF11 may be a potential therapeutic target in BRCA patients. CASP9 (caspase 9), a member of caspase family protease, has been identified with apoptotic and non-apoptotic roles, including autophagy regulation (30) and endosomal transport (31). GPX4 (glutathione peroxidase 4) is essential for maintaining redox homeostasis and protecting cells against lipid peroxidation. As a key peroxidation inhibiting protein, the activity of GPX4 is associated with a wide variety of diseases, including cancer and degenerative diseases (32–34). Proinflammatory cytokine IL18 (interleukin 18) is released through pores in the cell membrane during inflammasome-mediated pyroptosis (6). Nucleotide binding/leucine-rich repeat (NLR) family caspase-activation and recruitment domain (CARD) domain containing 4 (NLRC4) inflammasome activates CASP1 after exposure to intracellular bacteria, such as *S. typhimurium* (35), and NLRC4 activation is critical in defense against enteric pathogens and systemic pathogens (36). The toll/interleukin-1 receptor (TIR) domain containing adaptor protein (TIRAP) is an adapter molecule which has an elaborate role in inflammatory signaling pathways and host immune signaling (37). TNF (tumor necrosis factor) is a proinflammatory cytokine generated by various cells in response to a wide variety of immune stimuli and stress conditions (38). TNF-α/HMGB1 (high mobility group box 1) inflammation signaling pathway regulates pyroptosis during acute liver failure and kidney injury (39).

Up to now, molecular subtypes or immune subtypes (40) have been established to better understand the biological mechanisms and improve the prognosis in BRCA. Based on the gene expression profiling, BRCA can be classified into five intrinsic molecular subtypes, including luminal A, luminal B, HER2-enriched, basal-like, and claudin-low. The tumor biological behavior and response to medical therapy are distinct in these different molecular subtypes (41). Consisting with our finding, pyroptosis-related genes (such as CASP9, TIRAP, IL18, SCAF11, and NLRC4) have been studied in difference researches (29, 42–44). Compared with other studies, we firstly established a seven pyroptosis-related gene signature that linked inflammation with immune infiltration, and provided some new insights into the development of BRCA.

There are several limitations in our study. First of all, due to the limited clinical characteristics on patients available in public databases, we could not conduct subgroup analyses by stratifying for more factors. Second, the pyroptosis-related gene signature was constructed and then validated based on publicly available retrospective cohorts, lacking of validation of real clinical data. Finally, we only explored the expression and function of SCAF11in breast cancer cell lines with preliminary experiments. Thus, more fundamental experiments are needed to further explore the molecular mechanism of pyroptosis-related genes in BRCA.

## Conclusion

Collectively, our study systematically identified a unique prognostic signature comprised of 7 pyroptosis-related genes. The pyroptosis-related gene signature exhibited its predictive value in BRCA patients and could be a practical tool to facilitate personalized management. The potential roles in tumor microenvironment suggested that pyroptosis in combination with immunotherapy might be a potential therapeutic strategy for BRCA patients.

## Data Availability Statement

The datasets presented in this study can be found in online repositories. The names of the repository/repositories and accession number(s) can be found in the article/Supplementary Material.

## Author Contributions

LC and QY: acquisition of data. YY, ZL, FJ, and QH: analysis and interpretation of data. ZX: conception and design. LO, SW, CF, and YL: data curation. QY and ZX: development of methodology. QY and JP: writing the manuscript and revision of the manuscript. All authors contributed to the article and approved the submitted version.

## Funding

This study was supported by grants from the Changsha Natural Science Foundation (kq2007071), the Natural Science Foundation of Hunan Province (2021JJ30904), the horizontal project (1 43010100), and the Science and Technology Innovation Program of Hunan Province (2021RC3029).

## Conflict of Interest

The authors declare that the research was conducted in the absence of any commercial or financial relationships that could be construed as a potential conflict of interest.

## Publisher's Note

All claims expressed in this article are solely those of the authors and do not necessarily represent those of their affiliated organizations, or those of the publisher, the editors and the reviewers. Any product that may be evaluated in this article, or claim that may be made by its manufacturer, is not guaranteed or endorsed by the publisher.

## Supplementary Material

Supplementary Figure 1 GSEA enrichment analysis for IL-18, NLRC4, TIRAP, TNF in BRCA. **(A)** KEGG and **(B)** GO pathway analysis for IL-18 in BRCA. **(C)** KEGG and **(D)** GO pathway analysis for NLRC4 in BRCA. **(E)** KEGG pathway analysis for TIRAP in BRCA. **(F)** KEGG and **(G)** GO pathway analysis for TNF in BRCA.

Supplementary Table 1 33 pyroptosis-related genes.

Supplementary Table 2 7 pyroptosis-related genes with p less than 0.2 screened by univariate COX regression.

Supplementary Table 3 Detailed information of pyroptosis-related seven-gene signature.
