# Supplementary figures and images for: A Prognostic Signature Consisting of Pyroptosis-Related Genes and SCAF11 for Predicting Immune Response in Breast Cancer
Source: Front Med (Lausanne). 2022 May 12;9:882763. doi: 10.3389/fmed.2022.882763 (PMC9133489; doi:10.3389/fmed.2022.882763)

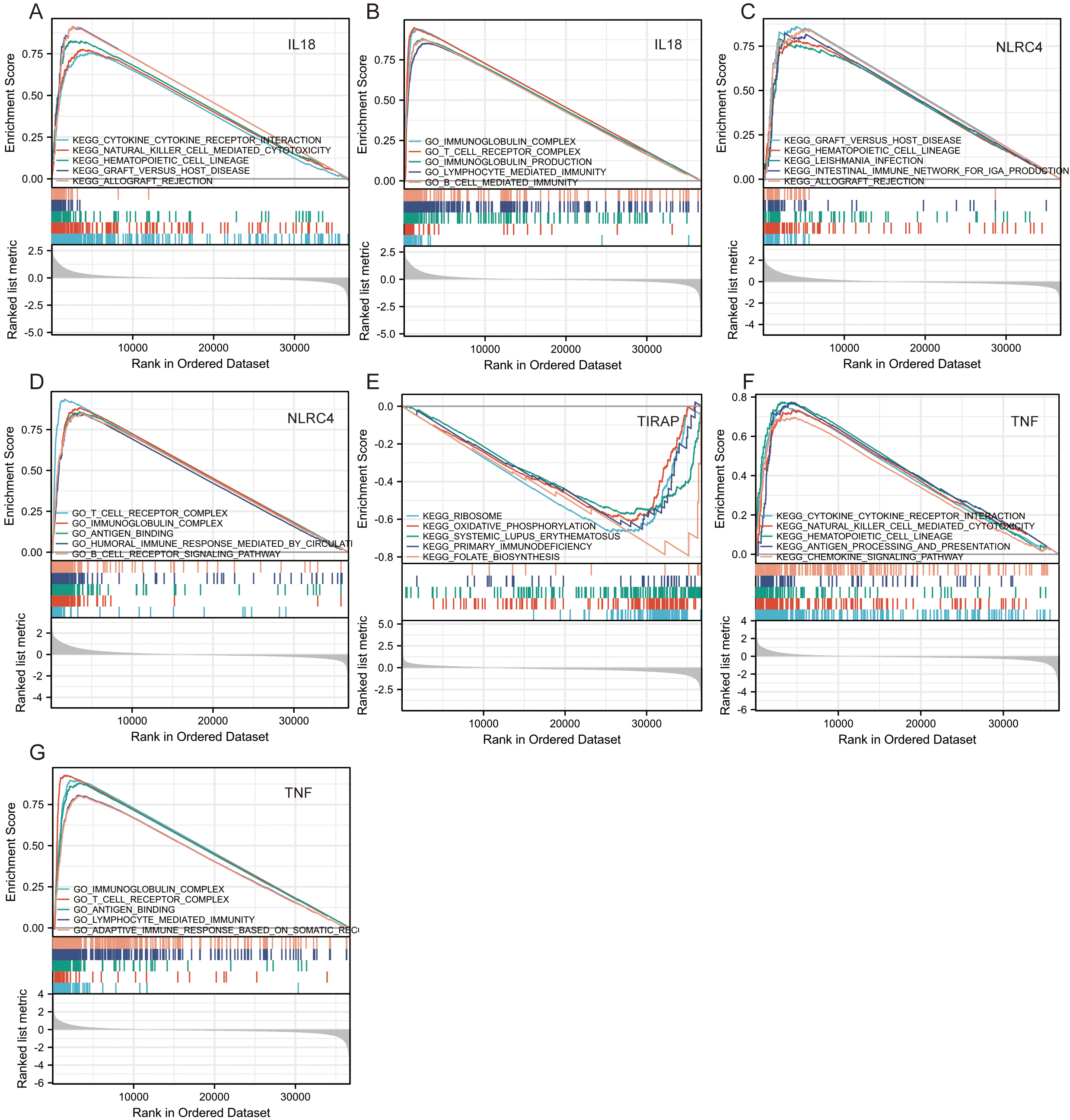

Supplement: Supplementary Figure 1 — GSEA enrichment analysis for IL-18, NLRC4, TIRAP, TNF in BRCA. (A) KEGG and (B) GO pathway analysis for IL-18 in BRCA. (C) KEGG and (D) GO pathway analysis for NLRC4 in BRCA. (E) KEGG pathway analysis for TIRAP in BRCA. (F) KEGG and (G) GO pathway analysis for TNF in BRCA. [file Image_1.TIF]
